# Manipulating and assembling metallic beads with Optoelectronic Tweezers

**DOI:** 10.1038/srep32840

**Published:** 2016-09-07

**Authors:** Shuailong Zhang, Joan Juvert, Jonathan M. Cooper, Steven L. Neale

**Affiliations:** 1 School of Engineering, University of Glasgow, Glasgow, G12 8LT, UK

## Abstract

Optoelectronic tweezers (OET) or light-patterned dielectrophoresis (DEP) has been developed as a micromanipulation technology for controlling micro- and nano-particles with applications such as cell sorting and studying cell communications. Additionally, the capability of moving small objects accurately and assembling them into arbitrary 2D patterns also makes OET an attractive technology for microfabrication applications. In this work, we demonstrated the use of OET to manipulate conductive silver-coated Poly(methyl methacrylate) (PMMA) microspheres (50 *μ*m diameter) into tailored patterns. It was found that the microspheres could be moved at a max velocity of 3200 *μ*m/s, corresponding to 4.2 nano-newton (10^−9^ N) DEP force, and also could be positioned with high accuracy via this DEP force. The underlying mechanism for this strong DEP force is shown by our simulations to be caused by a significant increase of the electric field close to the particles, due to the interaction between the field and the silver shells coating the microspheres. The associated increase in electrical gradient causes DEP forces that are much stronger than any previously reported for an OET device, which facilitates manipulation of the metallic microspheres efficiently without compromise in positioning accuracy and is important for applications on electronic component assembling and circuit construction.

The current technology to assemble discrete components into electronic circuits is mainly based on surface-mount technology (SMT), which uses a mechanical manipulator arm with a vacuum tip to pick and place the components on to target circuit board^1^. SMT is currently limited to components no smaller than 400 × 200 *μ*m (0402 metric) and this size limit comes from the minimum diameter allowed for a robust vacuum tip with the reliability required for picking up components. Alternative approaches are mandated by the ongoing drive towards assembling ever smaller components, allowing more functionality to be packed into portable devices such as mobile phones and smart watches. Previous research has examined the utility, for this application, of optical tweezers^2^, dielectrophoresis (DEP) based on patterned metal electrodes^3^,^4^, Langmuir-Blodgett assembly techniques^5^, atomic force microscopy probe^6^, and transfer printing technology^7^,^8^. However, to date, these techniques have relevant drawbacks; they cannot assemble micro-objects with a sufficiently wide range of sizes due to the limitation of exerted force^2^,^3^,^4^; they are typically unable to selectively manipulate large numbers of individual objects in parallel^3^,^4^,^5^; they are unable to perform highly accurate assembly^3^,^4^,^5^; they require complex and expensive positioning equipment^7^,^8^; they are unable to assemble objects with fragile structures due to the requirement of physically contacting and catching the targeted objects using stamp or tip^7^,^8^. So, at this point, cost-effective, compact, precise and efficient manipulation of individual or small groups of micro-objects with an extended size range still remains a challenge.

In this paper, we report the use of a new cost-effective assembly technology based on a touch-less opto-electro-fluidic technique known as optoelectronic tweezers (OET), which has the potential to perform parallel assembling of micro-objects with high accuracy. OET is an emerging technology that uses light-controlled DEP force to manipulate micro-objects such as cells^9^,^10^,^11^,^12^,^13^. Compared with conventional optical tweezers (OT) where particles are moved simply by passing momentum from light to the particle, OET traps have been found to be 470 times stiffer for a similar light intensity^10^ and capable of massive manipulation of cells and micro-particles in parallel (15,000 traps at once with just 1 mW of light)^9^. These advantages also make it an attractive approach for microfabrication applications. Previous OET studies have demonstrated the successful assembly of various nanoscale components, such as semiconductor nanowires, metallic nanowires and metallic spherical nanocrystals^14^,^15^,^16^,^17^,^18^. The metallic nanoscale objects can be used to produce conductive tracks and for interconnecting different components and creating conductive links^4^,^18^. However, to build up a circuit or an electronic device containing components with scales of one or several hundreds of microns, such as 0402 metric SMT components and recently-reported micro-lasers^19^, it is more desirable to use metallic objects with scales of several or several tens of microns to form conductive link as this cuts down the number of interfaces between conductive components reducing the chances of a poor connection and associated high resistance. In this work, we present the results of successful manipulation and assembly of large (50 micron diameter) silver-coated Poly(methyl methacrylate) (PMMA) microspheres by positive DEP force using an OET device. It was found that the microspheres can be moved at a max velocity of 3200 *μ*m/s, corresponding to a strong DEP force of 4.2 nano-newton (10^−9^ N). This velocity is the highest yet reported for using OET as a manipulation tool. Additionally, the microspheres can be positioned with high accuracy due to the strong DEP force (accuracy quantified by average positioning deviation in X direction: 0.4 ± 0.5 *μ*m; in Y direction: 0.2 ± 0.4 *μ*m). The magnitude of the DEP force in an OET device is usually proportional to the volume of the particle, the permittivity of the medium, the relative permittivity of the particle and the medium (the Clausius-Mossotti factor) and the gradient of the electric field squared^12^. The force we observed was much greater than those found before using dielectric particles and this can not be accounted for the Clausius-Mossotti factor which is limited to a range of −0.5 to 1 for spherical particles. Therefore, a finite element simulation was carried out to clarify the underlying mechanism and this suggests that the silver shells of the microspheres have strong influence on the surrounding electric field, which explains the observed strong DEP force in the experiment. The strong DEP forces facilitate manipulation of the metallic microspheres efficiently without compromise in positioning accuracy, which is important for applications on electronic component assembling and circuit construction.

## Results

### Background and experimental setup

**Figure 1:**
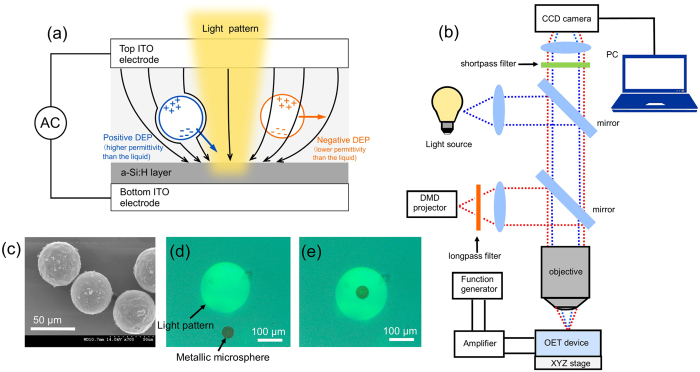
(**a**) Schematic diagram of an OET device. The chamber consists of two ITO electrodes with an AC voltage applied across them. The black arrows show the non-uniform electric field resulting from the resistivity change caused by the illumination of the a-Si:H photoconductive layer. The particle is attracted to the illuminated area by positive DEP force or pushed away by negative DEP force depending on its effective permittivity; (**b**) schematic experimental setup; (**c**) SEM image of silver-coated PMMA microspheres at 700x magnification; microscope images of a metal microsphere (**d**) before and (**e**) after being trapped by a 200 *μ*m diameter circular light pattern.

As shown in Fig. 1(a), a typical OET device consists of two electrodes made of indium tin oxide (ITO) coated glass slides which form a sample chamber. The bottom electrodes has a further thin coating of photoconductive semiconductor material, typically hydrogenated amorphous silicon (a-Si:H). A liquid buffer containing micro-particles is placed between the two electrodes, which are connected to a function generator. In the dark, the impedance of the photoconductive layer is very high and most of the voltage is dropped across it. Where a light pattern is imaged onto the photoconductive layer, the impedance of this layer drops significantly and the voltage is dropped into the liquid above the illuminated area, creating a non-uniform electrical field between the dark and illuminated regions. For the polarizable samples in the liquid, a dipole is induced in the object, which interacts with the non-uniform electrical field. When the object is more polarisable than the solvent it is attracted to the region of highest field (positive DEP force); while for an object of lower polarizability than the solvent, the object is pushed away from the region of highest field (negative DEP force). Therefore, by controlling the position of illumination regions in the OET device, ‘traps’ can be created to manipulate the position of samples.

The optical setup used in the experiment is shown in Fig. 1(b). As shown, the light pattern from the DMD projector (Dell 1510X) is introduced into the microscope (Olympus BX51, with motorised Prior Scan111 stage) and imaged onto the OET device, which is driven by the amplified signal from the function generator (TG5011 LX1 with amplifier Thurlby Thandor Instrument WA31). The camera is used to record experimental images and videos for further data analysis. The position of the OET device is controlled by the XY stage while the DMD and camera are kept stationary. A longpass filter (Thorlabs FD1R) is used to filter out the blue light from the projector and only the green-red portion of its output is projected onto the OET device. A shortpass filter (Thorlabs FES0550) is also used in front of the camera to filter out the strong red emission from the projector so that the image on the camera is not saturated and clear images and videos of the particles being manipulated can be recorded. The two electrodes used for the OET device are ITO-coated glass slides with an ITO thickness of 600 nm. The photoconductive layer used in the OET device is a-Si:H, which is deposited on top of ITO-coated glass slide at a thickness of 1 *μ*m by plasma-enhanced chemical vapour deposition (PECVD). The two electrodes were mounted together by a 150 *μ*m thick double sided tape to form a chamber. The metallic microspheres used in this work are silver-coated PMMA beads with diameters around 50 *μ*m (size range: 45 *μ*m to 53 *μ*m) and an average silver-shell thickness of 250 nm (Cospheric, PMPMS-AG-1.53). Figure 1(c) shows a Scanning Electron Microscopy (SEM) image of the silver-coated PMMA microspheres at 700x magnification. In this work, an average bead diameter of 50 *μ*m was used to calculate the DEP force. To make the sample for OET experiment, the microspheres were put into a solution containing deionized water and a volume ratio of 0.05% non-ionic surfactant ‘TWEEN 20’ (SIGMA P9416). Then, the solution containing metallic microspheres was pipetted into the chamber of the OET device. To provide electric potential across the OET device, a 10 kHz 25 V peak-to-peak AC voltage was used, which is similar to the bias conditions used to manipulate other silver nanoscale objects in OET devices^18^. After adding an AC voltage across the OET device and projecting a light pattern onto its photoconductive layer, the metallic microspheres were attracted to the illumination region due to positive DEP force, as shown in Fig. 1(d,e). In this work the heating effect was not taken into account for the following reasons. Firstly, the silver-coated beads trapped by light patterns did not move when the applied voltage was turned off, indicating the thermophoresis effect is not strong enough to move these beads. Secondly, the optical power density of the light spot to create traps was measured to be 0.65 W/cm^2^. From previously reported work^20^, if the optical power density is below 200 W/cm^2^, the DEP force will dominate over the electro-thermal force caused by light-induced heating of the photoconductive layer and the surrounding solution.

### Experimental results

**Figure 2:**
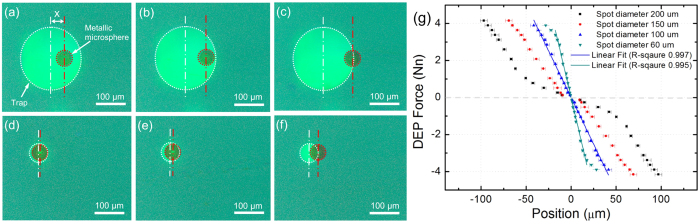
Microscope images of metallic microspheres trapped by 200 *μ*m diameter circular light pattern at (**a**) 600 *μ*m/s, (**b**) 1600 *μ*m/s and (**c**) 3200 *μ*m/s; microscope images of metallic microspheres trapped by 60 *μ*m diameter circular light pattern at (**d**) 600 *μ*m/s, (**e**) 1400 *μ*m/s and (**f**) 3000 *μ*m/s. The metallic microsphere is outlined in red and the trap created by the light pattern is outlined in white. X is the centre-to-centre distance between the microsphere and the trap, which are indicated by vertical lines. (**g**) Trap profiles of metallic microspheres created by 200 *μ*m, 150 *μ*m, 100 *μ*m and 60 *μ*m diameter light pattern.

Before measuring the trap profile of metallic microspheres in response to different light patterns, the Reynolds number for the microspheres moving at their maximum velocity in OET device was calculated (1.79 × 10^−4^), which suggested that they are manipulated in the laminar flow regime. Therefore the viscous drag force on the particle can be found from Stokes’ law and when the particle is dragged through the liquid by DEP this is equal to the DEP force^10^,^21^,^22^:


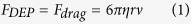


where *η* is the viscosity of the liquid, *r* is the radius of the microsphere and *ν* is velocity of the microsphere. The relative velocity of the microsphere and the light pattern is controlled by the motorised XY stage, which gradually increases to a velocity until the microsphere falls out of the trap. Figure 2(a–f) show the microscope images of metallic microspheres trapped by 200 *μ*m diameter and 60 *μ*m diameter circular light patterns at different velocities. As shown, the centre-to-centre distance between the microsphere and the light pattern increases as the velocity increases. A video of the metallic microsphere trapped by a 200 *μ*m diameter circular light pattern and moving at different velocities can be found in Supplementary Vidoe S1. By measuring the centre-to-centre distance between the trapped microsphere and circular light pattern at varying velocities, a trap profile can be plotted, which shows the DEP force experienced by a microsphere at different locations within the trap. It is worth mentioning that the silver-coated microspheres have large sizes (50 *μ*m diameter) and a density of 1.53 g/cm^3^, causing them to sediment to the surface of the a-Si:H. The light-induced DEP force also pulls down the microspheres to the bottom of the OET chamber. Therefore, an assumption was made that the microspheres sit in proximity of the a-Si:H surface due to gravity and DEP force. In this case, Faxen’s correction based on the radius of the microsphere (25 *μ*m) was used to calculate the DEP force^10^. To further clarify the influence of the distance between the bead and the substrate to the force, viscous drag force for a metallic bead moving at 3200 *μ*m/s was calculated using Faxen’s correction based on different distances between the bead centre and the a-Si:H surface (see results in Supplementary Fig. S1). As shown, although the force changes as the distance changes, the expected separation (less than a few hundred nanometers^23^) will have little effect on the forces calculated.

Figure 2(g) shows the trap profiles for metallic microspheres in circular light patterns with 200 *μ*m, 150 *μ*m, 100 *μ*m and 60 *μ*m diameter. The error bars come from the uncertainty in the measurement of the centre-to-centre distance between the microsphere and the light pattern. The metallic microspheres can be moved at a max velocity of 3200 *μ*m/s by traps created by light patterns with 200 *μ*m and 150 *μ*m diameters, corresponding to a DEP force of 4.2 nN, and a max velocity of 3000 *μ*m/s by traps created by light patterns with 100 *μ*m and 50 *μ*m diameters, corresponding to a DEP force of 3.9 nN. To the best of our knowledge, the velocity of the microsphere is the highest yet reported for using OET as a manipulation tool. Due to the limitations of different driving conditions and object sizes, it is difficult to make direct comparison between the DEP force of the metallic microspheres to that of widely reported metallic and semiconductor nanowires, PMMA microspheres, and cells^10^,^14^,^24^,^25^. However, it is worth mentioning that the metallic microspheres experience very high DEP force in the regime of several nano-newton (10^−9^ N), while all other previously reported micro-objects can only experience DEP force in the regime of several or several tens of pico-newton (10^−12^ N). Additionally, the metallic microspheres can experience DEP force even with very small displacements from the centre of large traps, e.g. a trap created by 200 *μ*m diameter light pattern, indicating large traps can also be used for fine control over the position of the metallic microsphere. This contrasts with previously reported results of using OET to manipulate HeLa cells^11^: the larger traps exhibit zero force on the cell for small displacements and only small traps similar to the size of the cell can be used for fine control over the position of the cell. The trap stiffness for metallic microspheres was also calculated using the gradient of a linear fit to the data. A good linear relationship between DEP force and displacement is hard to be achieved for large traps created by 200 *μ*m and 150 *μ*m diameter light patterns, meaning such traps have inconstant trap stiffness. However, constant trap stiffness can be achieved for smaller traps by good linear fitting of the data, as shown in Fig. 2(g). A trap stiffness of 1.0 × 10^−4^ N/m and 1.8 × 10^−4^ N/m was achieved for traps created by light patterns with 100 *μ*m and 60 *μ*m diameter, respectively. These are the stiffest traps created by using OET as a manipulation tool.

**Figure 3:**
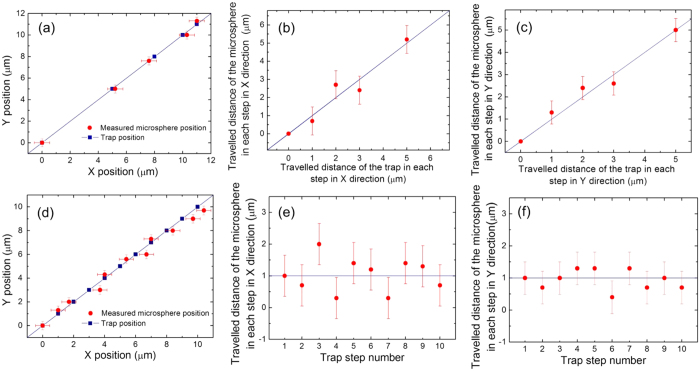
(**a**) Measured position of the metallic microsphere compared to the position of the trap. The stage is moved in steps of decreasing length (5 *μ*m, 3 *μ*m, 2 *μ*m, 1 *μ*m) in the direction with 45 degree to X axis and 45 degree to Y axis; (**b**) travel distance of the metallic microsphere in each step in X direction vs. that of the trap for the stage moved in steps of decreasing length (5 *μ*m, 3 *μ*m, 2 *μ*m, 1 *μ*m); (**c**) travel distance of the metallic microsphere in each step in Y direction vs. that of the trap for the stage moved in steps of decreasing length (5 *μ*m, 3 *μ*m, 2 *μ*m, 1 *μ*m); (**d**) Measured position of the metallic microsphere compared to the position of the trap. The stage is moved in successive steps of 1 *μ*m in the direction with 45 degree to X axis and 45 degree to Y axis; (**e**) travel distance of the metallic microsphere in each step in X direction vs. that of the trap for the stage moved in successive steps of 1 *μ*m; (**f**) travel distance of the metallic microsphere in each step in Y direction vs. that of the trap for the stage moved in successive steps of 1 *μ*m.

The strong DEP force and trap stiffness are very useful for the well-controlled manipulation of metallic microspheres. To test this we explored the positional accuracy achievable, Fig. 3(a–c) show the results of an experiment where the stage was moved in steps of decreasing length in the direction with 45 degree to X axis and 45 degree to Y axis. The first step was 5 *μ*m, followed by 3 *μ*m, 2 *μ*m and finally 1 *μ*m. Figure 3(a) shows the absolute position of the microsphere centre vs. that of a trap, which is created by a 60 *μ*m diameter light pattern. As shown, the measured microsphere position matches well with the position of the trap and follows the trend of the solid line, indicating the position of the microsphere can be well controlled by the trap. Since the resolution of camera is 3 pixels *μ*m^−1^, the error bars (X: ±0.5 *μ*m, Y: ±0.4 *μ*m) come from the uncertainty in the measurement of the distance. Figure 3(b,c) compares the travel distance of the microsphere to that of the trap in each step in X direction and Y direction, respectively. It can be observed that the travel distance of the microsphere is very close to the expected value in all the steps in both X and Y direction. Figure 3(d–f) show the results for the test where the stage is moved in successive steps of 1 *μ*m in the direction with 45 degree to X axis and 45 degree to Y axis. Figure 3(d) shows the absolute position of the microsphere compared to that of the trap. As in the previous experiment, ideally both the data sets should match up. Considering the error, the microspheres deviates from the expected position in no more than 1.3 *μ*m in X direction and no more than 1 *μ*m in Y direction in the worst possible case. Additionally, the average deviation from the expected position is 0.4 ± 0.5 *μ*m in X direction and 0.2 ± 0.4 *μ*m in Y direction which we believe is a good measure of the position accuracy in this case. Shown in Fig. 3(e,f) are the absolute travel distance in each step in X direction and Y direction, respectively. In this case, it should be always around 1 *μ*m. Again, once the error is considered, the travel distance would deviate from expected 1 *μ*m travel distance in no more than 1.7 *μ*m in X direction and no more than 1.2 *μ*m in Y direction in the worst case.

**Figure 4:**
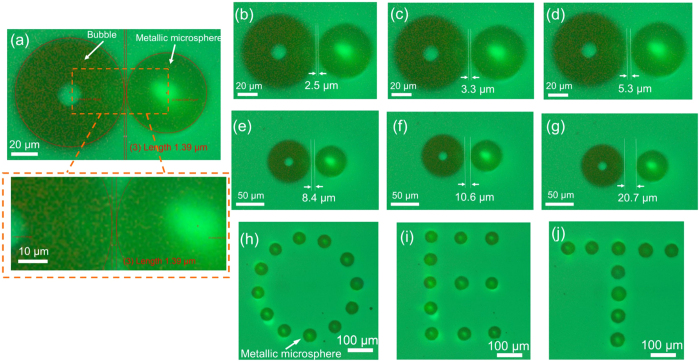
Microscope images of metallic microsphere and reference bubble with varied spacing: (**a**) 1.39 *μ*m, (**b**) 2.5 *μ*m, (**c**) 3.3 *μ*m, (**d**) 5.3 *μ*m, (**e**) 8.4 *μ*m, (**f**) 10.6 *μ*m and (**g**) 20.7 *μ*m. An example of the measurement lines (red coloured) fitted to the microsphere and bubble boundaries to estimate the spacing, is shown in (**a**). Microscope images of (**h**) ‘O’, (**i**) ‘E’, (**j**) ‘T’ formed by assembling the metallic microspheres in parallel via the OET device.

To further assess the high-accuracy positioning capabilities of the metallic microsphere in OET device, the metallic microsphere was positioned with varied spacing relative to a reference object. Figure 4(a–g) shows the microscope images of a metallic microsphere with varied separation to a bubble, starting from 1.39 *μ*m and increasing to 20.7 *μ*m. The metallic microsphere was positioned relative to the bubble using a pre-calculated set of coordinates in the stage control software, which were chosen to give varied target separations between the metallic microsphere and the bubble, starting from 1 *μ*m, followed by 2 *μ*m, 3 *μ*m, 5 *μ*m, 8 *μ*m, 10 *μ*m and finally 20 *μ*m. The microscope’s image analysis functions were used to measure the separations between the metallic microsphere and the bubble, specifically by superimposing best-fit circular ring to the boundaries of microsphere and bubble in close-up image acquisitions, as shown in Fig. 4(a). Each measurement was repeated ten times and the effective separation was thus calculated through averaging the acquired data and providing a measure of the random error introduced through this measurement technique. Numerical results corresponding to Fig. 4(a–g) and illustrating high degree of accuracy are summarized in Table 1. Deviations from the expected position were observed, which are below 1 *μ*m. The uncertainly in the measurement (random errors) mainly comes from the resolution of our system, as already noted, is 3 pixels *μ*m^−1^. Additionally, the microspheres do not have a perfect spherical shape, which influences the circular fitting of the microsphere shape and thus the measurement accuracy. Shown in Fig. 4(h–f) are microscope images of ‘O’ ‘E’ ‘T’ pattern formed by assembling the metallic microspheres in parallel via the OET device, which demonstrates the capability of using OET to arrange metallic microspheres into arbitrary patterns with high precision. This ability to precisely control the position of the metallic components is an important attribute when considering OET as an assembly technology. Future work in this area will focus on using the OET device to assemble conductive links made up by metallic microspheres and also to assemble SMT components including 0402 metric resistors and capacitors which have metallic components similar to silver-coated microspheres. Some pilot work of using OET to move and orientate a 0603 metric capacitor can be found in the Supplementary information (see Supplementary Fig. S2, Supplementary Fig. S3, and Supplementary Video S2). The results in this paper demonstrate that particles with metallic parts can experience strong DEP forces and be positioned with high accuracy, which make the OET device an ideal manipulation platform.

## Simulation and Discussion

**Figure 5:**
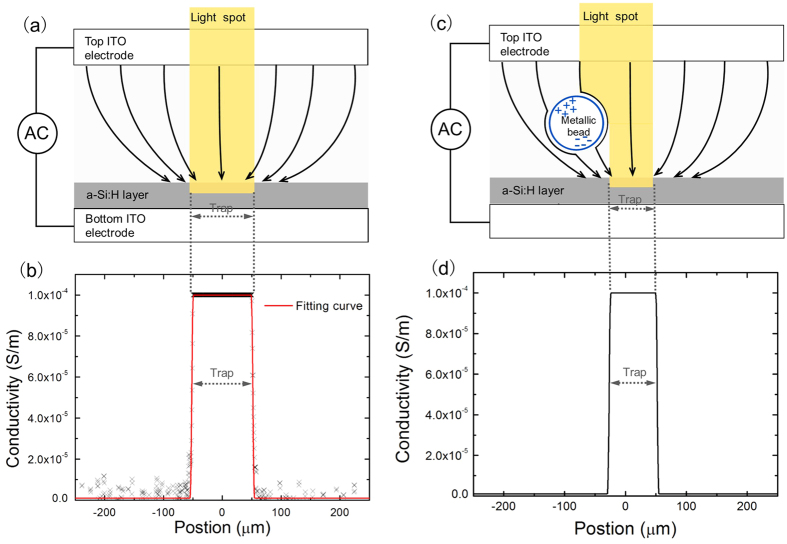
(**a**) Schematic diagram of an OET device, where a light spot is used to create the trap; (**b**) the conductivity profile of the a-Si:H as a function of position (under the illumination of a 100 *μ*m diameter light spot) and the fitting curve; (**c**) schematic diagram of an OET device with a metallic microsphere in the chamber. A light spot is used to create the trap and the metallic microsphere casts a shadow on the a-Si:H layer beneath it; (**d**) fitted conductivity of the a-Si:H where the shadow effect of a metallic microsphere (50 *μ*m diameter) at the left side of the trap edge (100 *μ*m diameter) is taken into consideration, reducing the width of the illuminated region.

Simulations were performed in the finite-element software COMSOL Multiphysics. The simulation model was similar to the 2D model reported previously^10^,^21^ and the simulation parameters were set according to the OET device used in the experiment and actual operating conditions. To simulate the case where 100 *μ*m diameter circular light spot was used to pattern the OET device, the light intensity profile of a 100 *μ*m diameter circular light spot was measured by recording its image and analysing it in Matlab. Figure 5(a) shows the schematic diagram of a light spot imaged on to an OET device. Since the image of the light spot was mainly projected in red, the red pixel value is used as a measure of the projected light intensity. To determine the conductivity of the illuminated and dark a-Si:H, the optical power density of the 100 *μ*m diameter light spot and the background illumination of a-Si:H were also measured. The optical power density of the light spot was measured to be 0.65 W/cm^2^, corresponding to a conductivity of 1 × 10^−4^ Sm^−1^ for the illuminated a-Si:H^20^; while the optical power density of the background illumination of the a-Si:H (dark a-Si:H) was measured to be 0.007 W/cm^2^, nearly two orders of magnitude smaller than that of the light spot. Since the conductivity of the a-Si:H increases linearly with the optical power density, the magnitude of the conductivity was taken at 1 × 10^−6^ Sm^−1^ for the dark a-Si:H^21^,^20^. In this case, the conductivity profile of the a-Si:H layer illuminated by a 100 *μ*m diameter light spot can be calculated based on its light intensity profile, as shown in Fig. 5(b). The conductivity profile of the 100 *μ*m diameter circular light spot was also fitted by a step-function distribution with continuous second derivative smoothing at the edge. This fitting curve was entered into the simulation model as the conductivity profile of the a-Si:H layer. In order to investigate the underlying physics of the strong DEP force exerted on metallic microspheres, simulation runs were performed to represent the case with and without the metallic microsphere at the edge of the trap. Since the metallic microsphere can reflect light and cast a shadow on the a-Si:H layer beneath it, the conductivity profile of a-Si:H layer needs to be modified to take the shadow effect into consideration, as shown in Fig. 5(c). Figure 5(d) shows fitted conductivity profile of the a-Si:H where the shadow effect of a metallic microsphere (50 *μ*m diameter) at the left side of the trap edge (100 *μ*m diameter) is taken into consideration. The conductivity profile shows a rise at −25 *μ*m due to the shadow effect of the metallic microsphere.

**Figure 6:**
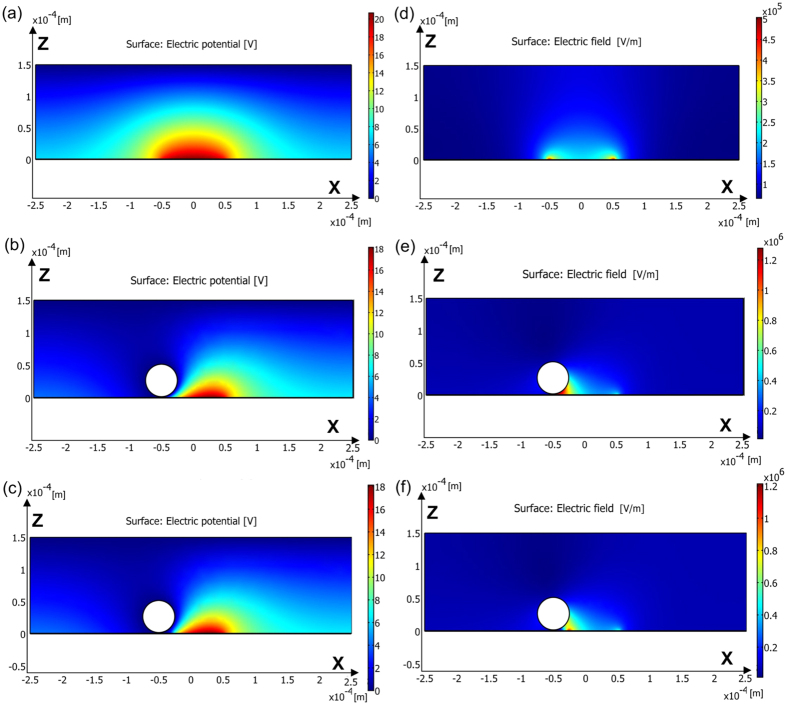
Simulation results of electric potential distribution for (**a**) OET device without metallic microsphere, (**b**) OET device with a metallic microsphere at the left edge of the trap but without shadow effect, and (**c**) OET device with a metallic microsphere at the left edge of the trap and with shadow effect. Simulation results of electric field distribution for (**d**) OET device without metallic microsphere, (**e**) OET device with a metallic microsphere at the left edge of the trap but without shadow effect, and (**f**) OET device with a metallic microsphere at the left edge of the trap and with shadow effect.

To make comparisons thus providing straightforward insights into the physical mechanism, three simulation runs were performed in this work: one without a metallic microsphere; one with a metallic microsphere at the left edge of the trap but no shadow effect; one with a metallic microsphere at the left edge of the trap and with shadow effect (thus one uses Fig. 5(d) as the conductivity profile). For the three simulation runs, the results of the electric potential distribution in the liquid above the a-Si:H are shown in Fig. 6(a–c), respectively. As shown for the case without a metallic microsphere in Fig. 6(a), the electric potential drop along the Z-axis is more prominent in the central region than in the edge, which is due to the difference in conductivity between the illuminated and dark a-Si:H. This simulation result is similar to previous reported work and suggests the OET device will work well due to the difference in electric potential distribution^21^. However, as shown in Fig. 6(b,c), when a metal coated bead is present there is a significant electric potential drop in the region between the metallic microsphere and the illuminated a-Si:H. To further analyse this phenomenon, the overall electric field distribution (containing electric field in both X and Z direction) was also calculated and shown in Fig. 6(d–f). For the case without metallic microsphere, the electric field distribution shown in Fig. 6(d) is quite similar to previously reported work^10^,^21^ showing two regions with strong electric fields at the edges of the trap where the conductivity of the a-Si:H sharply changes. However, in Fig. 6(e,f), strong electric fields appear in the region between the metallic microsphere and the illuminated a-Si:H, and the magnitude of the electric field in this region also significantly increases. Further analysis suggests this strong electric field is caused by the silver shell of the metallic microsphere. The electric field will propagate along this metallic surface and finally into the illuminated a-Si:H^26^. Therefore, the electric field between the metallic microsphere and the illuminated a-Si:H will increase accordingly, creating a large gradient of the electric field just next to the microsphere and hence a large DEP force. This simulation result is very different from the case where dielectric microspheres are manipulated by an OET device. Since the dielectric microspheres do not influence the external electric field significantly, the electric field distribution in an OET device with and without the dielectric microspheres will be similar to when they are present. In Fig. 6(e), the max electric field appears in the region about −50 *μ*m to the centre of the trap while in Fig. 6(f) the max electric field appears in the region about −25 *μ*m from the centre of the trap. This difference is due to different conductivity profiles of the a-Si:H for the two simulation runs, with and without the shadow effect, as the max electric field normally appears in the boundary region between dark and illuminated a-Si:H, where the conductivity significantly changes.

**Figure 7:**
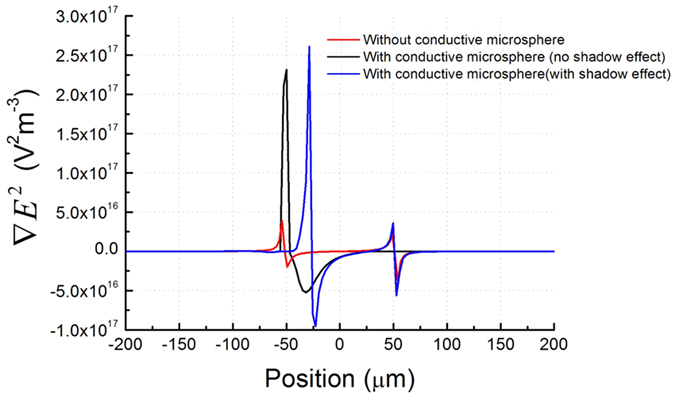
Gradient of the electric field squared in the liquid just above the a-Si:H for the three simulation runs. OET device without a metallic microsphere (red line), OET device with a metallic microsphere at the left edge of the trap but without the shadow effect (black line), and OET device with a metallic microsphere at the left edge of the trap and with the shadow effect (blue line).

Figure 7 shows the gradient of the electric field squared in the liquid 0.5 *μ*m above the a-Si:H for the three simulation runs. For the cases with a conductive microsphere in the OET device, the peak of the gradient of the electric field squared appears at different positions for the simulation run with shadow effect and the one without shadow effect. This is due to the different conductivity profiles of the a-Si:H for the two simulation runs. Compared with the simulation without a conductive microsphere, the gradient of the electric field squared in the simulation with metallic microsphere shows much stronger increase in the region between dark and illuminated a-Si:H (peak of the gradient of the electric field squared increases by 6.7 times and 5.9 times for the cases with and without shadow effect), indicating there is a significant change of the electric field in this region due to the influence of a metallic microsphere. A simple analysis of the DEP force can be made by considering a single dipole interacting with the electric field and is proportional to the gradient of the electric field squared, i.e. *F*_*DEP*_ ∝ ∇*E*^2^ 12,26. In reality as the field varies greatly around the microsphere there will be a more complex multi-poled force however these simulation results suggest the strong DEP force experienced by metal coated microspheres is due to the increase of the electric field and the increase of the gradient of the electric field squared caused by the metallic microsphere in the OET device. This effect then differs from previously reported studies of dielectric particles in OET devices. The metallic microspheres can modify the electric field distribution in the OET device significantly, which is very different from dielectric microspheres such as PMMA and glass microspheres^24^,^25^. Since the metallic microsphere can not be well represented by a single dipole and the gradient of electric field squared varies significantly across the metallic microsphere, the traditional way of calculating the DEP force based on the dipole moment and average gradient of the electric field squared is not applicable. Our 2D simulation model shows how the electric field changes in an OET device with metallic microspheres, which provides reasonable explanation of the observed experimental results. However, to calculate the DEP force of the metallic microsphere and clarify the influence of shadow effect to the DEP force, a 3D simulation model is more desirable. We are currently developing a 3D simulation model and calculating the DEP force of the metallic microsphere based on this 3D model and are using the Maxwell stress tensor over the surface of the microsphere which then takes the variation into the gradient into account^27^.

## Conclusion

In summary, we have demonstrated the use of a positive DEP force to manipulate and assemble silver-coated PMMA microspheres in an OET device. It was found that the metallic microspheres could be moved at a max velocity of 3200 *μ*m/s, corresponding to 4.2 nano-newton (10^−9^ N) DEP force, which enables the microspheres to be manipulated with high accuracy and reliability. Simulations were also carried out to explain the unexpectedly strong forces observed in the experimental results and it was found that the strong DEP force exerted on metallic microsphere was due to the significant increase of the electric field in the OET device due to the influence of the silver shells of the microspheres on the fields in the device. This work is important for applications in electronic component assembling and circuit construction using OET and also insightful for using OET to manipulate micro-objects with similar metallic properties.

## Methods

### Device fabrication

The OET device used in this work consists of a top electrode and a bottom electrode. The top electrode is made from a standard microscope glass slide (2.5 cm × 7.5 cm) coated on one side with 600 nm thick ITO. The ITO coating was deposited by magnetron sputtering (Diamond Coatings Ltd, UK). On preparation of the top electrode, the ITO-coated glass slide was cut to approximately 2.5 cm × 1.5 cm using a diamond scribe and then bonded to an electrical wire using conductive silver paint (Agar Scientific, Acheson Silver DAG 1415M). After the silver paint dried out, the electrical wire was further glued using an epoxy resin (Mxbon Waterproof Epoxy-E41A). The bottom electrode was also made from the same ITO-coated glass slide. However, an extra photoconductive layer was deposited on top of the ITO-coated glass slide. This photoconductive layer is made of a-Si:H, which was deposited at a thickness of 1 *μ*m by plasma-enhanced chemical vapour deposition (PECVD) from a pure silane gas at 300 °C with 10 W input power and 10 mTorr pressure for 59 minutes. On preparation of the bottom electrode, the ITO-coated glass slide with a-Si:H layer was also cut to a size of approximately 2.5cm × 1.5 cm. Then, approximately 2 mm^2^ of the a-Si:H at the edge was removed by a scratch knife to expose the conductive ITO layer, where an electrical wire was mounted using silver paint and epoxy as for the top electrode. After the top and bottom electrodes were fabricated, they were attached together by a 150 *μ*m thick double sided tape to form a chamber, where the solution containing metallic microspheres can be injected via pipette. This attached electrode pair formed the OET device used in the experiment.

### Sample preparation

The metallic microspheres used in this work are commercially-available silver-coated PMMA beads (Cospheric, PMPMS-AG-1.53), which are in powder formats as provided from the company. To make the sample for OET experiment, the metallic microspheres were added to a solution, consisting of deionized water with 0.05% volume ratio of non-ionic surfactant ‘TWEEN 20’ (SIGMA P9416). Since the metallic microspheres have a tendency to ‘clump together’ in unorganised complexes and stick onto the surface of pipette, adding ‘TWEEN 20’ in the solution can minimise the clumps and help transfer the microspheres into the OET chamber. The conductivity of the sample was measured to be 2 mSm^−1^ after adding the TWEEN 20. To carry out the experiment, the solution containing the metallic microspheres was injected into the chamber of the OET device at a volume of 10 *μ*L using a pipette.

### Numerical simulation

COMSOL Multi-physics 3.5a (COMSOL Inc., USA) finite element software was used to calculate the electric field distribution and the gradient of the electric field in the OET device. To set up the simulation model, an AC/DC module (In-Plane Electric Currents) was used. This module uses the quasi-static approximation which assumes that the device is much smaller compared to the wavelength of the applied AC signal^21^,^25^. This assumption is justified in this case as the area of the device modelled is 500 *μ*m across in X plane and 150 *μ*m across in Z plane, and the wavelength of the applied AC signal is 3 × 10^4^ m. The boundary conditions were chosen as electrical insulation at the sides of the model to represent the mainly vertical electrical field. A continuation boundary was set for all interior boundaries whilst the top boundary was set to 0 V and the bottom boundary set to 20 V to simulate the applied AC signal.

## Additional Information

**How to cite this article**: Zhang, S. *et al*. Manipulating and assembling metallic beads with Optoelectronic Tweezers. *Sci. Rep*. **6**, 32840; doi: 10.1038/srep32840 (2016).

## Supplementary Material

Supplementary Information

Supplementary Movie S1

Supplementary Movie S2

## Figures and Tables

**Table 1:** Comparison between the targeted (software input) microsphere and bubble spacing and that obtained after positioning the microsphere, averaged from ten measurements (error bar comes from standard deviation of the measured data).

Targeted (*μ*m)	Measured (*μ*m)
1.0	1.4 (±0.4)
2.0	2.5 (±0.4)
3.0	3.3 (±0.4)
5.0	5.3 (±0.4)
8.0	8.4 (±0.4)
10.0	10.6 (±0.4)
20.0	20.7 (±0.4)
