# Supplementary material for: Manipulating and assembling metallic beads with Optoelectronic Tweezers
Source: Sci Rep. 2016 Sep 7;6:32840. doi: 10.1038/srep32840 (PMC5013433; doi:10.1038/srep32840)
Supplement: Supplementary Information [file srep32840-s1.doc]

Supplementary information:

Manipulating and assembling metallic beads with optoelectronic tweezers

Shuailong Zhang*****, Joan Juvert, Jonathan M. Cooper and Steven L. Neale*****

School of Engineering, University of Glasgow, Glasgow, *G12 8LT, UK*

******Corresponding author*: [Shuailong.Zhang@glasgow.ac.uk](mailto:Shuailong.Zhang@glasgow.ac.uk)

**Supplementary figures:**


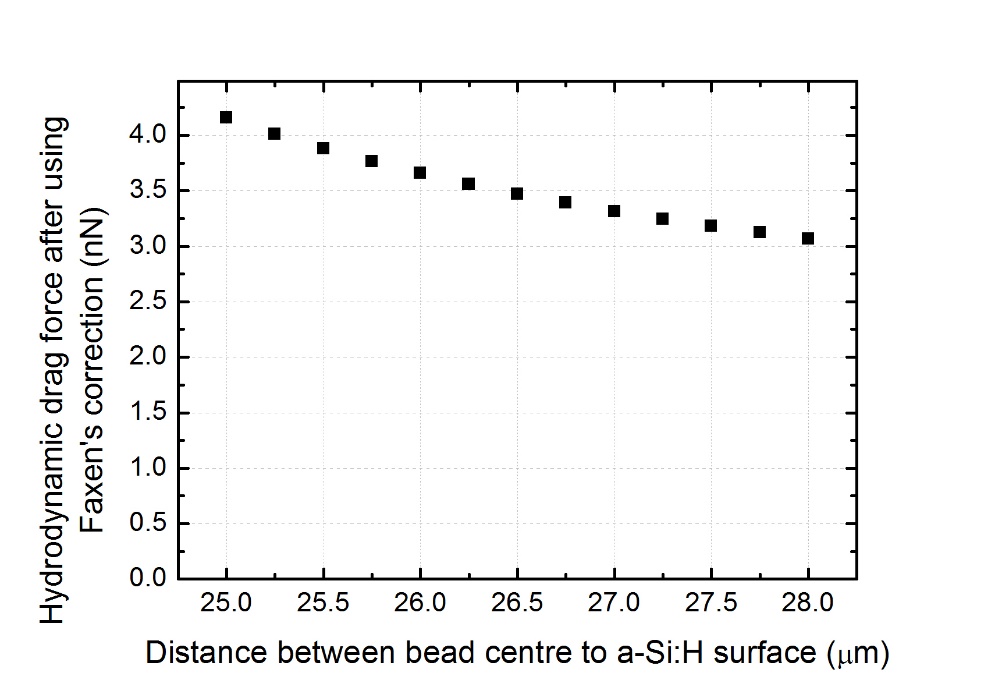


**Figure S1.** Hydrodynamic drag force for a silver-coated Poly(methyl methacrylate) (PMMA) bead (50 µm diameter) moving at a speed of 3200 µm/s in deionised water using Faxen’s correction based on different distances between the bead centre and the hydrogenated amorphous silicon (a-Si:H) surface.

As shown, although the drag force changes as the distance from the particle to the surface changes, there would be little effect from a separation of less than a few hundred nanometers which is the expected gap between the component and the surface of the device [1]. Therefore, the conclusion of this work that the metallic bead can feel large DEP force in the regime of several nano newton is reasonable.


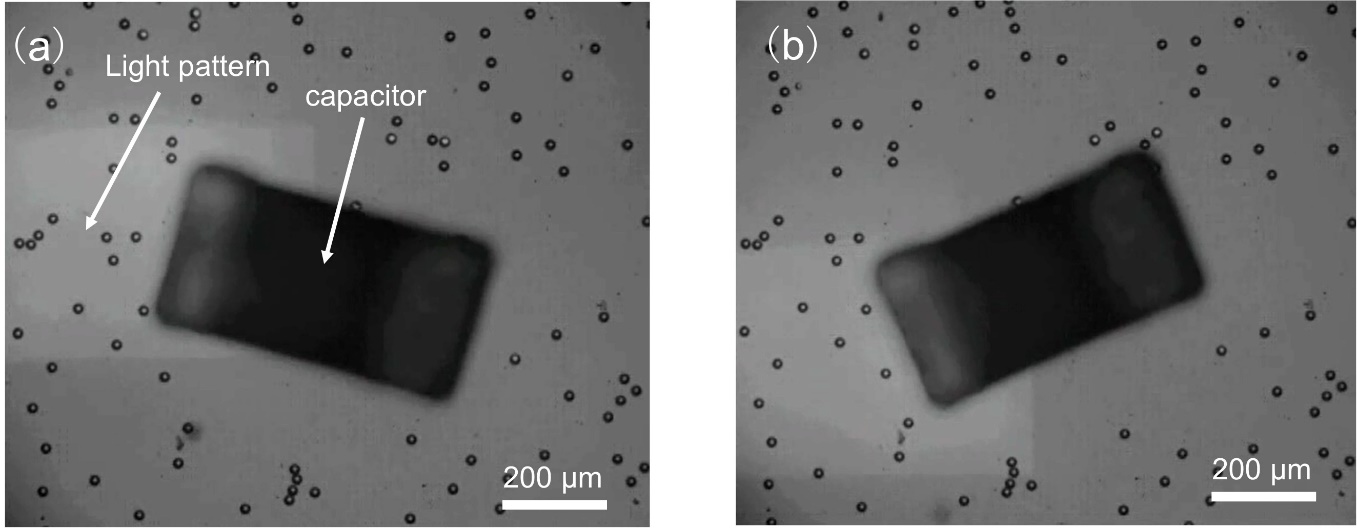


**Figure S2.** A 0603 metric surface-mount-technology (SMT) capacitor (a) before and (b) after being moved in optoelectronic tweezer (OET) device. Some PMMA beads are put into the OET device as reference objects. The conditions used were an applied bias of 30Vpp at 10kHz in 5mSm^-1^ buffer solution containing 0.025% surfactant (TWEEN20, Sigma-Aldrich).


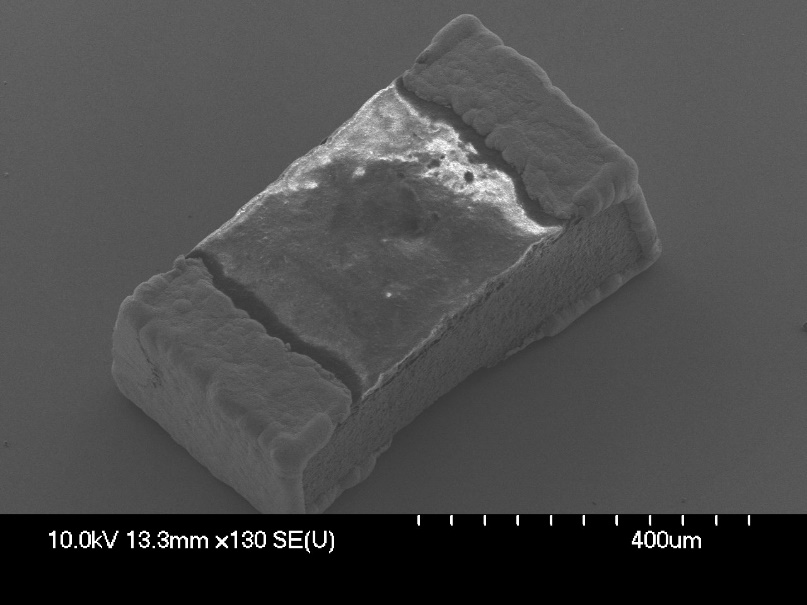


**Figure S3.** Scanning electron microscope image of a 0603 metric SMT capacitor at 130x magnification. As shown, this capacitor has a metal pad at each end of the device

**Supplementary Video**

**Video S1:** A metallic bead is trapped by a 200 µm diameter circular light pattern and moved at different velocities (400 µm/s, 800 µm/s, 1600 µm/s, 2400 µm/s, and 3200 µm/s).

**Video S2:** A 0603 metric SMT capacitor is moved and orientated by a light pattern in OET device.

**References**

[1] Kremer, C. *et al.* Shape-Dependent Optoelectronic Cell Lysis. [*Angewandte Chemie*](http://eprints.gla.ac.uk/view/journal_volume/Angewandte_Chemie_=28International_Edition=29.html) **53**, 861-865 (2014).
